# Variables that predict hospital stay and the outcome of Fournier gangrene at King Abdulaziz University Hospital: a retrospective study

**DOI:** 10.1186/s12894-024-01496-7

**Published:** 2024-05-16

**Authors:** Bandar Alhubaishy, Omar M. Bahassan, Abdulrahman E. Alsabban, Ali H. Alkhzaim, Ziyad A. Alnefaie, Kamal S. Algarni, Sultan G. Almehmadi, Saud N. Alqahtani

**Affiliations:** Urology Department, King Abdulaziz University Hospital, Jeddah, Saudi Arabia

**Keywords:** Predict, LHS, Outcome, Fournier, Gangrene, KAUH

## Abstract

**Background:**

The aggressive nature of Fournier gangrene and the associated health issues can result in a more complex clinical course and potentially a longer hospital stay. This study aimed to assess factors that affect the length of hospital stay (LHS) and its relation to the outcome of Fournier gangrene patients.

**Methods:**

A retrospective study was performed at King Abdulaziz University Hospital (KAUH), Saudi Arabia, on patients diagnosed with Fournier gangrene between 2017 and 2023. Data about length of hospital stay (LHS), age, BMI, clinical and surgical data and outcome was obtained.

**Results:**

The mean age of the studied patients was 59.23 ± 11.19 years, the mean body mass index (BMI) was 26.69 ± 7.99 kg/m^2,^ and the mean duration of symptoms was 10.27 ± 9.16 days. The most common presenting symptoms were swelling or induration (64%), 88% had comorbidities with diabetes mellitus (DM) (84%), and 76% had uncontrolled DM. of patients, 24% had a poly-microbial infection, with *E. coli* being the most common (52%). The mean length of hospital stay (LHS) was 54.56 ± 54.57 days, and 24% of patients had an LHS of more than 50 days. Longer LHS (> 50 days) was associated with patients who did not receive a compatible initial antibiotic, whereas shorter LHS was associated with patients who received Impenem or a combination of vancomycin and meropenem as alternative antibiotics following incompatibility. Reconstruction patients had significantly longer LHS and a higher mean temperature. However, none of the studied variables were found to be predictors of long LHS in the multivariate regression analysis.

**Conclusion:**

Knowledge of the values that predict LHS allows for patient-centered treatment and may be useful in predicting more radical treatments or the need for additional treatment in high-risk patients. Future multicenter prospective studies with larger sample sizes are needed to assess the needed variables and predictors of long LHS.

## Introduction

Fournier gangrene, a rare but potentially life-threatening condition, is a rapidly progressing and aggressive form of necrotizing fasciitis that affects the genital, perineal, and perianal regions. Named after the French surgeon Jean-Alfred Fournier, who first described the condition in 1883 [1].

Fournier gangrene is characterized by the destructive spread of infection through soft tissues, leading to severe tissue damage, necrosis, and potential systemic complications. While it predominantly affects adult males, it can also occur in females and children [2].

Rapid diagnosis, immediate medical intervention, and surgical debridement are crucial to prevent its progression and improve patient outcomes [3].

Over time, there has been an evident evolution in the clinical manifestation of Fournier gangrene. This progression has been marked by a rising incidence of occurrences among individuals presenting with underlying comorbidities [4, 5].

These comorbidities, which may include chronic diseases such as diabetes mellitus, obesity, immunosuppression, and cardiovascular disorders, have emerged as significant contributing factors to the development and progression of Fournier gangrene [6].

The interaction between these underlying health issues and the aggressive infection characteristic of Fournier gangrene can lead to a more complex clinical course and potentially poorer outcomes [2, 7].

Despite advancements in medical care and early intervention strategies, mortality rates in Fournier gangrene remain notable. Consequently, a scoring system has been formulated to assist in predicting patient prognosis. The Fournier Gangrene Severity Index (FGSI), created by Laor and colleagues, serves as a tool to evaluate the potential for mortality in individuals afflicted with Fournier gangrene [8].

This study aimed to assess factors that affect the length of hospital stay (LHS) and its relation to the outcome of Fournier gangrene patients.

## Subjects and methods

This was a retrospective study performed at King Abdulaziz University Hospital (KAUH), Saudi Arabia, from May to August 2023. The inclusion criteria were patients diagnosed with Fournier gangrene between 2017 and 2023 in the study setting. The diagnosis and inclusion of the patient were based on the physical examination with the presence of erythema, subcutaneous crepitation, skin discolouration and necrosis.

A predesigned checklist was prepared to collect data about patients’ LHS, age, BMI, symptom duration, presenting symptoms, comorbidity, risk factors, laboratory results, culture results, initial antibiotics and compatibility, inpatient surgical intervention, lesion location, site of skin debridement, inpatient complications, reconstruction and outcome.

The mean length of hospital stay (LHS) was 54.56 ± 54.57, thus the participants were divided into two categories (≤ 50 days and > 50 days) based on the mean LHS. The comparison between the two groups was performed according to patient’ age, BMI, symptom duration, presenting symptoms, comorbidities, risk factors, culture results, initial antibiotics and their compatibility, inpatient surgical intervention, lesion location, site of skin debridement, inpatient complications, reconstruction, outcome and laboratory results.

The patients’ condition information was taken from emergency department data based on the patient’s history, clinical examination of the patients, and investigations. There is no specific hospital policy for this group of patients. The choice of empiric antibiotics was determined by the evaluation and recommendations of the infectious diseases team. Empiric antibiotic therapy was initiated for patients with septic shock while awaiting the results of sensitivity and specificity testing. Septic shock was diagnosed during this waiting period.

Data were statistically analysed using SPSS version 26. To investigate the association between the variables, the chi-squared test (χ2) was applied to qualitative data that were expressed as numbers and percentages. The association between the quantitative nonparametric variables that were expressed as the mean and standard deviation (mean ± SD) was examined using the Mann‒Whitney test. The odds ratio (OR) was calculated at a confidence interval (CI) of 95% to assess the risk factors (independent predictors) of prolonged LHS (> 50 days) among the studied patients in the multivariate logistic regression analysis. A p value of less than 0.05 was regarded as statistically significant.

## Results

**Fig. 1:**
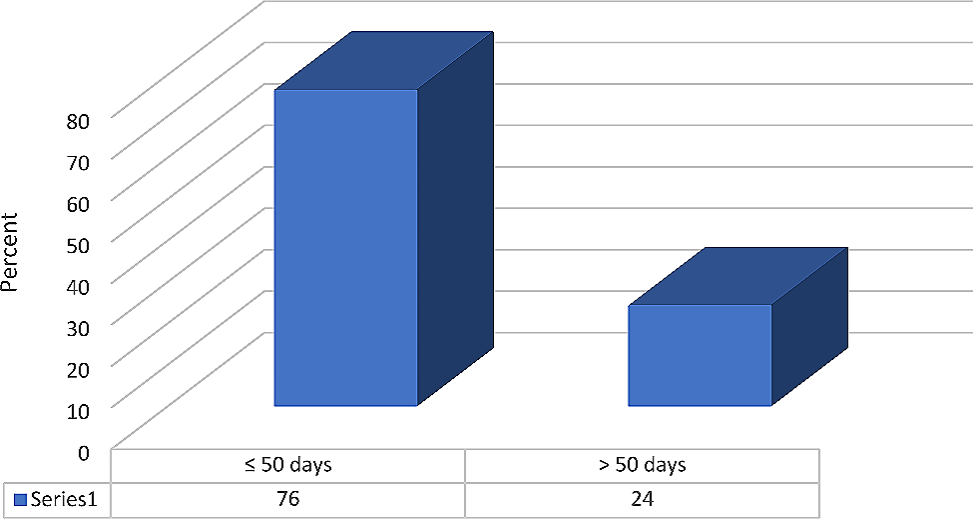
Percentage distribution of LHS durations

Of the 25 studied patients, all patients were males with no history of trauma, and all of them underwent skin debridement. The mean length of hospital stay (LHS) was 54.56 ± 54.57 days. Figure 1 shows that 6 patients (24%) had LHS for more than 50 days.

Table 1 shows that the mean age of the studied patients was 59.23 ± 11.19 years, the mean BMI was 26.69 ± 7.99 kg/m^2,^ and the mean duration of symptoms was 10.27 ± 9.16 days. The most common presenting symptoms were swelling or induration (64%) and pain (60%). Of the patients, 22 (88%) had comorbidities, with DM (84%) being the most common. Most patients had risk factors (88%), with uncontrolled DM (76%) and immobilization (24%) being the most common. Of the patients, 24% had a poly-microbial infection, and the most common organism that was revealed in the culture analysis was *E. coli* (52%). Patients with a longer LHS (> 50 days) had a significantly higher percentage of *Pseudomonas aeruginosa*-positive cultures (*p* ≤ 0.05). A nonsignificant relationship was found between LHS and patient age, BMI, symptom duration, presenting symptoms, comorbidity or risk factors (p = > 0.05).

**Table 1:** Relationship between LHS and patient age, BMI, symptom duration, presenting symptoms, comorbidities, risk factors and culture results (No.: 25)

Variable	Total No. (%)	LHS		χ2	*p* value
		≤ 50 days	> 50 days		
**Age (years)**	59.23 ± 11.19	57.63 ± 11.95	64.67 ± 6.4	1.36*	0.185
**BMI (kg/m2)**	26.69 ± 7.99	26.83 ± 8.99	26.29 ± 4.39	0.89*	0.923
**Symptoms duration (**days**)**	10.27 ± 9.16	11.63 ± 10.37	6.67 ± 2.87	0.37*	0.407
**Presenting symptoms**					
Pain	15 (60)	11 (57.9)	4 (66.7)	0.14	0.702
Erythema	7 (28)	7 (36.8)	0 (0.0)	3.07	0.08
Swelling or induration	16 (64)	13 (68.4)	3 (50)	0.67	0.412
Fever	10 (40)	6 (31.6)	4 (66.7)	2.33	0.126
Purulent discharge	9 (36)	7 (36.8)	2 (33.3)	0.02	0.876
**Comorbidity**	22 (88)	17 (89.5)	5 (83.3)	0.16	0.867
If having a comorbidity, specify: (No.:22)					
DM	21 (84)	16 (84.2)	5 (83.3)	0.003	0.959
HTN	13 (52)	10 (52.6)	3 (50)	0.01	0.91
Obesity	3 (12)	2 (10.5)	1 (16.7)	0.16	0.687
CVD	5 (20)	4 (21.1)	1 (16.7)	0.05	0.815
ESRD	3 (12)	3 (15.8)	0 (0.0)	1.07	0.299
Other	12 (48)	9 (47.4)	3 (50)	0.01	0.91
**Risk factors**	22 (88)	17 (89.5)	5 (83.3)	0.16	0.867
Smoking	4 (16)	3 (15.8)	1 (16.7)	0.003	0.959
Uncontrolled DM	19 (76)	14 (73.7)	5 (83.3)	0.23	0.629
Immobilization	6 (24)	6 (31.6)	0 (0.0)	2.49	0.114
Immunocompromised	5 (20)	4 (21.1)	1 (16.7)	0.05	0.815
Old age	5 (20)	4 (21.1)	1 (16.7)	0.05	0.815
**Culture results**					
Poly-microbes	6 (24)	5 (27.8)	1 (16.7)	0.29	0.586
Single microbe	19 (76)	13 (72.2)	5 (83.3)		
**Organism type**					
*E. coli*	13 (52)	9 (47.4)	4 (66.7)	0.68	0.409
Yeast cells	2 (8)	2 (10.5)	0 (0.0)	0.68	0.407
*Staphylococcus aureus*	2 (8)	2 (10.5)	0 (0.0)	0.68	0.407
*Pseudomonas aeruginosa*	2 (8)	0 (0.0)	2 (33.3)	6.88	**0.009**
K. pneumonia	2 (8)	2 (10.5)	0 (0.0)	0.68	0.407
Other	4 (16)	2 (21.1)	0 (0.0)	1.5	0.22

N.B. : * = Mann–Whitney test

Table 2 demonstrates that the most commonly used initial antibiotic was combined with tazobactam (48%), and for 72% of patients, the initial antibiotic was compatible. For those with noncompatible initial antibiotics, the most common alternative was Imipenem. More than half of the patients (56%) had inpatient surgical intervention, and the most common lesion location was the genital location (88%). It was observed that patients with a shorter LHS (≤ 50 days) had a significantly higher percentage of those with compatible initial antibiotics and a higher percentage of those who received imipenem or combined vancomycin and meropenem as alternative antibiotics after noncompatibility (*p* ≤ 0.05).

**Table 2:** Relationship between LHS and initial antibiotics and their compatibility, inpatient surgical intervention and lesion location (No.: 25)

Variable	Total No. (%)	LHS		χ2	*p* value
		≤ 50 days	> 50 days		
**Initial antibiotics**					
Ceftriaxone	2 (8)	2 (10.5)	0 (0.0)	4.44	0.488
ceftriaxone and metronidazole	1 (4)	1 (5.3)	0 (0.0)		
Cefuroxime + Metronidazole	1 (4)	0 (0.0)	1 (16.7)		
Meropenem	8 (32)	6 (31.6)	2 (33.3)		
Metronidazole	1 (4)	1 (5.3)	0 (0.0)		
Tazocine	12 (48)	9 (47.4)	3 (50)		
**The initial antibiotics were compatible**	18 (72)	16 (84.2)	2 (33.3)	5.85	**0.016**
**If initial antibiotics not compatible, what antibiotic was given**: (No.:7)					
ciprofloxacin	1 (4)	0 (0.0)	1 (16.7)	15.18	**0.019**
ciprofoxacin + vancomycin	1 (4)	0 (0.0)	1 (16.7)		
Impenem	2 (8)	2 (10.5)	0 (0.0)		
piperacillin/Tazobactam	1 (4)	0 (0.0)	1 (16.7)		
vancomycin and meropenem	1 (4)	1 (5.3)	0 (0.0)		
vancomycin and clindamycin	1 (4)	0 (0.0)	1 (16.7)		
**Inpatient surgical intervention**	14 (56)	10 (52.6)	4 (66.7)	0.56	0.754
Colostomy	6 (24)	3 (15.8)	3 (50)	2.92	0.087
Suprapubic tube	3 (12)	1 (5.3)	2 (33.3)	3.4	0.065
**Lesion location**					
perianal	12 (48)	9 (47.4)	3 (50)	0.01	0.91
genital	22 (88)	16 (84.2)	6 (100)	1.07	2.99
gluteal	6 (24)	6 (31.6)	0 (0.0)	2.49	0.114
others	5 (20)				
**If other location, specify: (No.: 5)**					
anterior abdomen	1 (20)	0 (0.0)	1 (16.7)	4.44	0.218
lower abdomen	1 (20)	1 (5.3)	0 (0.0)		
upper thigh	3 (60)	3 (15.8)	0 (0.0)		

**Fig. 2:**
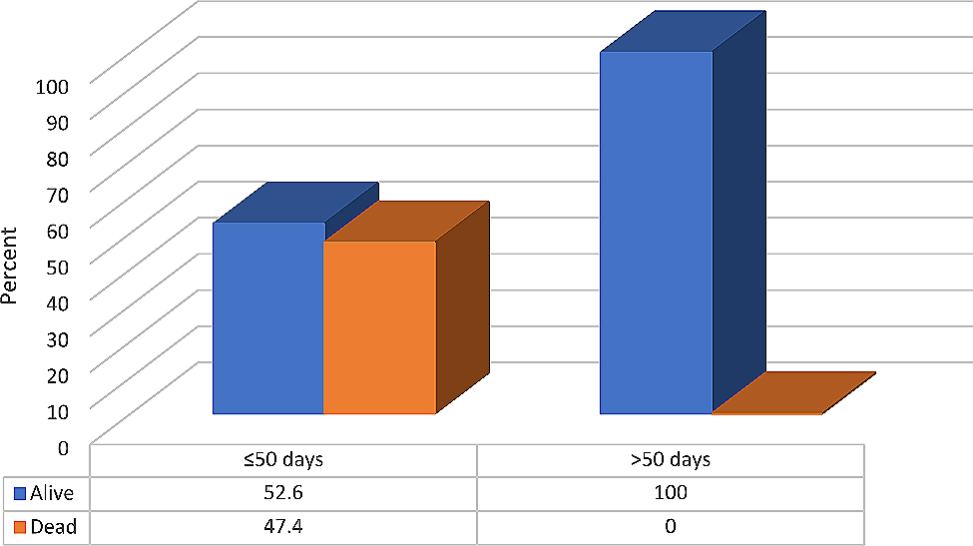
Relationship between LHS and patient outcomes (No.: 25). N.B.: (χ2 = 4.44, *p*-value = 0.035)

Table 3; Fig. 2 illustrate that the most common site of skin debridement was the scrotum (88%). Of the patients, 11 (44%) had inpatient complications, with septic shock (32%) being the most common complication. One-fifth (20%) of patients had reconstruction, and 9 (36%) died. It was found that patients with a longer LHS (> 50 days) had a significantly higher percentage of reconstruction and a significantly higher percentage of those who were alive (p = < 0.05).

**Table 3:** Relationship between LHS and site of skin debridement, inpatient complications, reconstruction and outcome (No.: 25)

Variable	Total No. (%)	LHS		χ2	*p* value
		≤ 50 days	> 50 days		
**Site of skin debridement**					
Scrotal	22 (88)	16 (89.5)	5 (83.3)	0.16	0.687
Penile	7 (28)	4 (21.1)	3 (50)	1.89	0.169
Peroneal	9 (36)	7 (36.8)	2 (33.3)	0.02	0.876
Lower abdomen	4 (16)	4 (21.1)	0 (0.0)	1.5	0.22
Upper thigh	5 (20)	5 (26.3)	0 (0.0)	1.97	0.16
Suprapubic	1 (4)	1 (5.3)	0 (0.0)	0.32	0.566
**Inpatient complications**	11 (44)	10 (52.6)	1 (16.7)	2.39	0.122
If having inpatient complication, specify: (No.:11)					
Infection	2 (8)	1 (5.3)	1 (16.7)	0.8	0.369
septic shock	8 (32)	8 (42.1)	0 (0.0)	3.71	0.054
**Reconstruction**	5 (20)	2 (10.5)	3 (50)	4.44	**0.035**
**Patients’ outcome**					
Alive	16 (64)	10 (52.6)	6 (100)	4.44	**0.035**
Dead	9 (36)	9 (47.4)	0 (0.0)		

N.B. (χ2 = 4.44, p value = **0.035)**

The mean values of the laboratory results of the studied patients are illustrated in Table 4. Patients who had longer LHS (> 50 days) had a significantly higher mean temperature than patients who had an LHS ≤ 50 days (p = < 0.05).

**Table 4:** Relationship between the LHS and mean values of laboratory results (No.: 25)

Variable	Mean SD	LHS		Mann‒Whitney test	*p* value
		≤ 50 days	> 50 days		
HgA1C	8.67 ± 2.02	8.43 ± 1.91	9.22 ± 2.33	0.44	0.783
FGSI (more or equal or less than 9	5.32 ± 3.82	5 ± 3.6	6.33 ± 4.67	0.46	0.737
Temp	37.14 ± 0.72	36.93 ± 0.57	37.78 ± 0.82	2.82	**0.01**
RR	22.96 ± 5.16	22.74 ± 4.82	23.8 ± 6.87	0.15	0.879
HR	108.63 ± 21.05	107.89 ± 22.4	111.4 ± 16.54	0.74	0.325
Serum sodium (mmol)	130 ± 4.24	130 ± 4.63	130 ± 3.03	0.29	0.32
serum K (mmol)	4.32 ± 0.84	4.36 ± 0.84	4.12 ± 0.91	0.36	0.72
Serum cr (mg)	183.36 ± 226.5	190.26 ± 353.26	161.5 ± 120.86	1.01	0.308
hematocrit	34.23 ± 8.75	33.58 ± 8.96	36.28 ± 8.47	0.65	0.522
WBCS mmx1000	20.03 ± 10.07	20.4 ± 11.12	18.87 ± 6.27	0.12	0.899

Multivariate logistic regression analysis was performed to assess the risk factors (independent predictors) of prolonged LHS among the studied patients. None of the variables that were significant in the univariate analysis (*Pseudomonas aeruginosa* infection, noncompatible initial antibiotics, reconstruction, high temperature) were found to be risk factors (independent predictors) of prolonged LHS (> 50 days) (Table 5).

**Table 5:** Multivariate logistic regression analysis of risk factors for prolonged LHS among studied patients

Variable	B	Wald	*p* value	Odds Ratio (CI:95%)
*Pseudomonas aeruginosa*	1.02	2.19	0.084	0.87 (1.03–2.98)
The initial antibiotics were compatible	1.74	0.13	0.192	0.14 (0.13–1.98)
Reconstruction	0.17	0.63	0.172	0.52 (0.22–1.45)
Temperature	3.03	1.96	0.161	0.33 (1.64–1.67)

## Discussion

Fournier gangrene is considered a disease with a high mortality rate [6]. Predisposing factors affecting the course of the disease are an important key factor in the progression of the disease, and identifying those elements can prevent the fulminant course of the disease and can lower the mortality rate of such disease.

All of the patients in the current study were men. An earlier study discovered a male/female ratio of 5.2/1 [9]. Males were found to be ten times more likely than females to contract the disease [10]. According to some studies, this significant difference is due to the drainage system of the female pelvic anatomy as women perineal region may better drain through vaginal secretions [10, 11]. The male predominance is consistent with previous findings reported in other studies [11, 12]. However, it was found that female patients with FG have greater BMI but similar clinical presentation, microbiologic characteristics and mortality rate compared to men [13].

The mean length of hospital stay (LHS) of studied patients in the present study was 54.56 ± 54.57 days. This LHS is longer than that observed in a previous study, where the average length of hospital stay was 13 days [14].

The average age of the patients studied was 59.23 11.19 years, with no relationship between age and LHS. This age range is consistent with previous research, which found an age range of 30–60 years [15]. Another study found that the majority of cases were between the ages of 50 and 60 [16]. Ghnnam [17] conducted a study in Egypt and found that the average age of patients at diagnosis was 51 years (21–72 years). Other studies [18, 19] reported an older age at presentation.

A study performed by Eksi et al., 2022, found a mean age of 55.1 ± 7.6 years and that age had no effect on LHS [9]. The same nonsignificant relationship between patient age and LHS was observed in other studies [20].

A multicenter study found that patients over 60 years of age were at risk for prolonged hospitalization, and the length of hospital stay was found to be proportional to an increase in the number of comorbidities [21]. In a study of 80 patients, Eksi et al. [9] discovered that age and gender had no effect on the length of hospital stay, which is consistent with the current findings.

The most common comorbidity among the patients studied was diabetes (88%), and the most common risk factor was uncontrolled diabetes (76%). Diabetes has been identified as a risk factor in 32–66% of FG cases but has been shown to have no effect on outcomes or mortality [22, 23].

Diabetes mellitus was the most commonly reported comorbid disease associated with this pathology, consistent with other studies [24–26]. Diabetes mellitus is estimated to affect 50 to 70% of Fournier’s gangrene patients by some authors [24, 26]. Diabetes mellitus has been identified as a risk factor for Fournier’s gangrene, with a more progressive and fatal outcome due to decreased phagocytic and intracellular bactericidal activity, as well as neutrophil dysfunction [25].

In the current study, a nonsignificant relationship was discovered between comorbidities as risk factors and LHS. A previous study found that the most common comorbidity in FG was diabetes, but no significant correlation could be found between diabetes and any other comorbidity and LHS [9]. However, in a previous study [27]. The presence of DM and HT, as well as the number of comorbidities, increased the length of hospital stay. In the Chalya et al. study [28], advanced age (> 60 years) and diabetes were associated with prolonged LHS.

Longer LHS (> 50 days) in the current study was significantly higher among patients who were alive. However, other studies found a significant difference between LOS and mortality rates [29–31].

This study found that the mean length of hospital stay (LHS) among studied patients was 54.56 ± 54.57 days. Shorter LHS was observed in previous studies [27, 32]. However, previous studies revealed that the reported length of hospital stay ranges from 2 to 276 days [20].

Morbidity and prolonged hospitalization remain significant issues because patients are frequently in their forties and fifties and have comorbidities. Multiple debridement procedures, reconstructive surgeries, diverting stoma procedures, and related complications are common in these cases, and hospitalization can last up to 9 months [27]. Several bacterial organisms, both aerobes and anaerobes, have been identified as agents that work together to cause the disease [33].

In the current study, 24% of patients had a polymicrobial infection, and the most common organism revealed in the culture analysis was *E. coli* (52%). Jiménez-Pacheco et al. discovered polymicrobial infection in 59.5% of cases [32].

Most experts believe that the polymicrobial nature of Fournier gangrene is needed to create the synergy of enzyme production that promotes the infection’s rapid multiplication and spread [34–36]. Our study’s microbiological results are consistent with the literature, with *Escherichia coli* being the most common organism [33]. Chalya et al. discovered that *E. coli* (28.3%) was the most common bacterial organism isolated [28].

The current study discovered a nonsignificant relationship between the site of skin debridement and LHS. Previous studies [37] discovered the same thing. At the same time, a retrospective study conducted in Turkey between 2013 and 2018 found no significant correlation between the Fournier Gangrene Severity Index (FGSI) and either the duration of hospital stay or the frequency of surgical debridement [38].

Treatment is based on early and extensive debridement to remove infected and necrotic tissue, hemodynamic stabilization, and broad spectrum antibiotics, according to the literature [39, 40].

The antibiotic regimen varies according to the center and antibiotic resistance in the geographic area where the microorganisms are isolated. Recent research suggests beginning empirical therapy with third-generation cephalosporins for gram-negative agents and metronidazole for anaerobes, with the possibility of adding aminoglycosides [39, 41].

Shorter LHS (50 days) was found to be significantly associated with receiving a compatible initial antibiotic and imipenem or combined vancomycin and meropenem as alternative antibiotics after incompatibility. When FG is diagnosed, broad-spectrum parental antibiotic therapy is administered empirically and then tailored based on culture results. Antibiotics must be effective against staphylococcal, streptococcal, and gram-negative bacteria, coliforms, Pseudomonas, Bacteroides, and Clostridium [42]. Empirical triple antibiotic therapy consists of a broad-spectrum penicillin or third-generation cephalosporin, an aminoglycoside (e.g., gentamicin), and metronidazole or clindamycin [13, 42].

Early diagnosis, broad-spectrum antimicrobial treatment, and prompt surgical debridement are the recommended treatments for Fournier’s gangrene [35, 42].

This study found a long mean LHS among studied patients. Longer LHS (> 50 days) was associated with patients who did not receive a compatible initial antibiotic, whereas shorter LHS was associated with patients who received imipenem or combined vancomycin and meropenem as alternative antibiotics after incompatibility. Patients who had reconstruction had significantly longer LHS and a higher mean temperature. Patients with FG had a long LHS. Knowing the values that predict LHS allows for patient-centered treatment and may be useful in predicting more radical treatments or the need for additional treatment in high-risk patients. Future multicenter prospective studies that include larger samples are needed to assess the needed variables and the predictors of long LHS.

### Limitations

A limitation of the present study was the small sample size. The retrospective single centre nature was another limitation that hinders the generalization of the study results. Another limitation was not using the Fournier Gangrene Severity Index (FGSI).

## Conclusion

Understanding the values that predict LHS enables patient-centered care and may help anticipate the need for more intensive therapies or follow-up care for high-risk patients. Larger sample sizes and further multicenter prospective studies are required to evaluate the necessary characteristics and predictors of extended LHS. It is recommended to use the Fournier Gangrene Severity Index (FGSI) in these studies.

## Data Availability

The datasets used and/or analyzed during the current study are available from the corresponding author upon reasonable request.

## Abbreviations

FG: Fournier gangrene
FGSI: Fournier Gangrene Severity Index
LHS: Length of hospital stay
KAUH: King Abdulaziz University Hospital
BMI: Body mass index
DM: Diabetes millets
SPSS: Statistical Backage of Social Software.
χ^2^: Chi-squared test
SD: Standard deviation
OR: Odds ratio
CI: Confidence Interval
